# 2-(3,4-Di­meth­oxy­phen­yl)-4-(thio­phen-2-yl)-2,3-di­hydro-1,5-benzo­thia­zepine

**DOI:** 10.1107/S1600536813034612

**Published:** 2014-01-08

**Authors:** B. C. Manjunath, M. Manjula, K. R. Raghavendra, S. Shashikanth, K. Ajay Kumar, N. K. Lokanath

**Affiliations:** a Department of Studies in Physics, Manasagangotri, University of Mysore, Mysore 570 006, India; b Department of Studies in Chemistry, Manasagangotri, University of Mysore, Mysore 570 006, India; c Post Graduate Department of Chemistry, Yuvaraja’s College, University of Mysore, Mysore 570 006, India

## Abstract

In the title compound, C_21_H_19_NO_2_S_2_, the seven-membered thia­zepine ring adopts a slightly distorted twist boat conformation. The dihedral angle between the benzene rings is 67.4 (2)°. The mean plane of the thio­phene ring is twisted by 59.3 (2) and 87.7 (2)° from the mean planes of the benezene rings. In the crystal, inversion dimers linked by pairs of C—H⋯O hydrogen bonds generate *R*_2_^2^(20) loops.

## Related literature   

For the biological activity of benzo­phenone derivatives, see: Cutignano *et al.* (2003 ▶); Sanjeeva *et al.* (2008 ▶). For a related structure, see: Manjula *et al.* (2013 ▶).
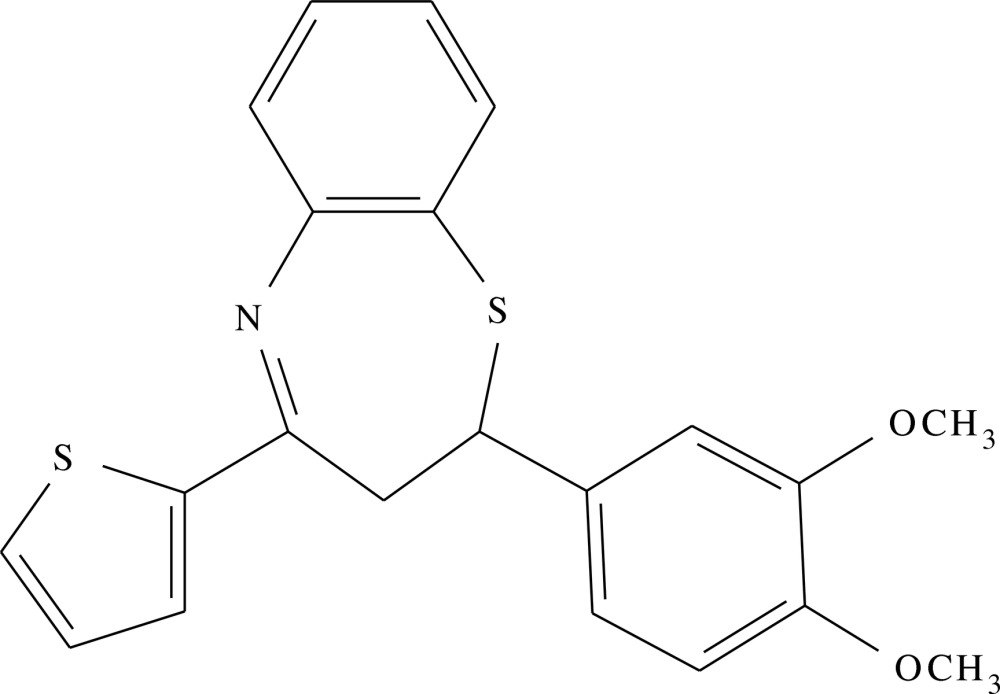


## Experimental   

### 

#### Crystal data  

C_21_H_19_NO_2_S_2_
*M* *_r_* = 381.51
Triclinic, 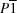

*a* = 8.6188 (11) Å
*b* = 9.7463 (15) Å
*c* = 11.9018 (16) Å
α = 100.308 (10)°
β = 107.921 (9)°
γ = 95.163 (11)°
*V* = 924.6 (2) Å^3^
*Z* = 2
Cu *K*α radiation
μ = 2.73 mm^−1^
*T* = 296 K
0.21 × 0.20 × 0.20 mm

#### Data collection  

Bruker X8 Proteum diffractometer
Absorption correction: multi-scan (*SADABS*; Bruker, 2013 ▶) *T*_min_ = 0.598, *T*_max_ = 0.611
7560 measured reflections
2999 independent reflections
2093 reflections with *I* > 2σ(*I*)
*R*_int_ = 0.051

#### Refinement  

*R*[*F* ^2^ > 2σ(*F* ^2^)] = 0.058
*wR*(*F* ^2^) = 0.180
*S* = 1.07
2999 reflections
237 parameters
H-atom parameters constrained
Δρ_max_ = 0.29 e Å^−3^
Δρ_min_ = −0.39 e Å^−3^

### 

Data collection: *APEX2* (Bruker, 2013 ▶); cell refinement: *SAINT* (Bruker, 2013 ▶); data reduction: *SAINT*; program(s) used to solve structure: *SHELXS97* (Sheldrick, 2008 ▶); program(s) used to refine structure: *SHELXL97* (Sheldrick, 2008 ▶); molecular graphics: *Mercury* (Macrae *et al.*, 2006) ▶; software used to prepare material for publication: *Mercury*.

## Supplementary Material

Crystal structure: contains datablock(s) global, I. DOI: 10.1107/S1600536813034612/hb7177sup1.cif

Structure factors: contains datablock(s) I. DOI: 10.1107/S1600536813034612/hb7177Isup2.hkl

Supporting information file. DOI: 10.1107/S1600536813034612/hb7177Isup3.cml

CCDC reference:

Additional supporting information:  crystallographic information; 3D view; checkCIF report

## Figures and Tables

**Table 1:** Hydrogen-bond geometry (Å, °)

*D*—H⋯*A*	*D*—H	H⋯*A*	*D*⋯*A*	*D*—H⋯*A*
C4—H4⋯O23^i^	0.93	2.54	3.457 (5)	169

Symmetry code: (i) 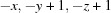.

**Fig. 1.**
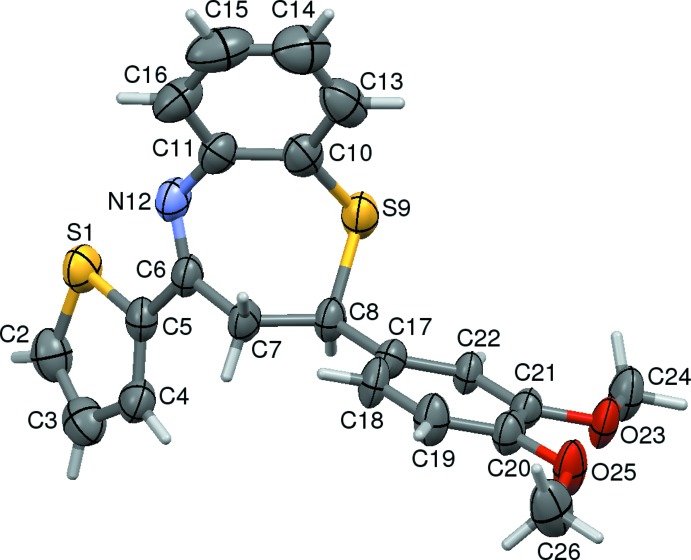
ORTEP diagram of the title molecule with 50% probability ellipsoids.

**Fig. 2.**
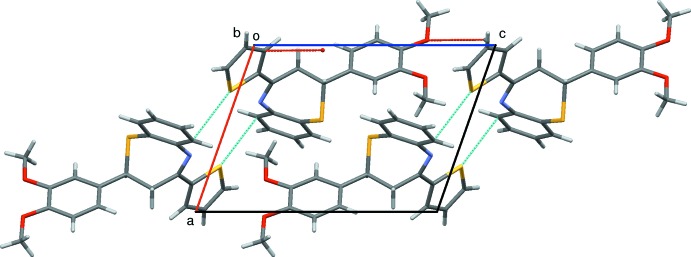
Packing diagram of molecule, viewed along the crystallographic b axis. Dotted lines represents intermolecular hydrogen bonding.

## supplementary crystallographic information

### 1. Comment

Seven membered ring compounds are receiving significant attention because of the existence of their structural units in some natural products (Cutignano *et al.*, 2003). Heterocycles containing the 1,4-thiazepine ring is one of important moieties in nitrogen and sulfur containing heterocycles and has been widely used as key building block for pharmaceutical agents as well as biologically active compounds. Benzothiazepine and its derivatives show a wide spectrum of pharmacological activities such as antifeedent, coronary vasodilatory, tranquilizer, antidepressant, *CNS* stimulant, antihypertensive, calcium channel blocker, antiulcer, calcium antagonist and antimicrobial agents (Sanjeeva *et al.*, 2008). The C6—N12 is shorter than an usual C—N single bond [1.294 Å compared to 1.416 Å] and the C10—S9 bond is shorter than an usual C—S single bond [1.760 Å compared to 1.82 Å]. The bond lengths and angles do not show large deviations and are comparable with those reported for a similar structure (Manjula *et al.*, 2013). The atoms C7, C8, C10 and C11 present in the central thiazepine ring forms a basal plane and the S9 atom as the bow, representing the boat conformation of thiazepine ring.

In the title compound, the dihedral angle between the mean planes of the benzene rings is 67.40°. The mean plane of the thiazepine ring is twisted by 36.17° and 77.93° from the mean planes of two benezene rings.

### 2. Experimental

A mixture of 2-aminothiophenol (4 mmol) with 3-(3,4-dimethoxyphenyl)-1- (thiophen-2-yl)prop-2-en-1-one (4 mmol) and 3–4 drops of conc. Hydrochloric acid in methanol (10 ml) was heated with stirring at 473 K for 4 h. The reaction was monitored by TLC (hexane/chloroform). After the completion of the reaction, the mixture was extracted in to ether (30 ml), washed successively with cold and dilute hydrochloric acid and water. The solvent was evaporated to dryness, the solid obtained was crystallized from 95° ethyl alcohol solution to get pale yellow needles of the title compound in 90° yield.

### 3. Refinement

All hydrogen atoms were located geometrically with C—H = 0.93–0.97) Å and allowed to ride on their parent atoms with *U*_iso_(H) = 1.2*U*_eq_(aromatic C).

### Crystal data

**Table:**

C_21_H_19_NO_2_S_2_	*Z* = 2
*M**_r_* = 381.51	*F*(000) = 400
Triclinic, *P*1	*D*_x_ = 1.370 Mg m^−^^3^
Hall symbol: -P 1	Cu *K*α radiation, λ = 1.54178 Å
*a* = 8.6188 (11) Å	Cell parameters from 2999 reflections
*b* = 9.7463 (15) Å	θ = 4.0–64.8°
*c* = 11.9018 (16) Å	µ = 2.73 mm^−^^1^
α = 100.308 (10)°	*T* = 296 K
β = 107.921 (9)°	Needle, light yellow
γ = 95.163 (11)°	0.21 × 0.20 × 0.20 mm
*V* = 924.6 (2) Å^3^	

### Data collection

**Table:**

Bruker X8 Proteum diffractometer	2999 independent reflections
Radiation source: Bruker MicroStar microfocus rotating anode	2093 reflections with *I* > 2σ(*I*)
Helios multilayer optics monochromator	*R*_int_ = 0.051
Detector resolution: 10.7 pixels mm^-1^	θ_max_ = 64.8°, θ_min_ = 4.0°
φ and ω scans	*h* = −10→9
Absorption correction: multi-scan (*SADABS*; Bruker, 2013)	*k* = −11→9
*T*_min_ = 0.598, *T*_max_ = 0.611	*l* = −7→13
7560 measured reflections	

### Refinement

**Table:**

Refinement on *F*^2^	Primary atom site location: structure-invariant direct methods
Least-squares matrix: full	Secondary atom site location: difference Fourier map
*R*[*F*^2^ > 2σ(*F*^2^)] = 0.058	Hydrogen site location: inferred from neighbouring sites
*wR*(*F*^2^) = 0.180	H-atom parameters constrained
*S* = 1.07	W = 1/[Σ^2^(*F*O^2^) + (0.0632*P*)^2^ + 0.4716*P*] WHERE *P* = (*F*O^2^ + 2*F*C^2^)/3
2999 reflections	(Δ/σ)_max_ < 0.001
237 parameters	Δρ_max_ = 0.29 e Å^−^^3^
0 restraints	Δρ_min_ = −0.39 e Å^−^^3^

### Special details

**Table:**

Experimental. m.p. 371 K. 1H NMR (CDCl3): δ 2.1 (d, 2H, C3—H), 3.80 (t, 1H, C2—H), 3.82 (s, 6H, -2OCH3), 6.52 (d, 1H, Ar—H), 6.64 (s, 1H, Ar—H), 7.0 (d, 1H, Ar—H), 7.1 (t, 1H, C5—H 5 m ring), 7.2–7.4 (m, 4H, Ar—H), 7.18 (t, 1H, C4—H 5 m ring), 7.46 (d, 1H, C3—H 5 m ring). Anal. Calcd. for C21H19NO2S2: C 66.11, H 5.02, N 3.67°; found C 66.10, H 4.98, N 3.68°. Mass FAB+ (NBA): 382 (*M* + 1, 100°).
Geometry. Bond distances, angles *etc*. have been calculated using the rounded fractional coordinates. All su's are estimated from the variances of the (full) variance-covariance matrix. The cell e.s.d.'s are taken into account in the estimation of distances, angles and torsion angles
Refinement. Refinement on *F*^2^ for ALL reflections except those flagged by the user for potential systematic errors. Weighted *R*-factors *wR* and all goodnesses of fit *S* are based on *F*^2^, conventional *R*-factors *R* are based on *F*, with *F* set to zero for negative *F*^2^. The observed criterion of *F*^2^ > σ(*F*^2^) is used only for calculating -*R*-factor-obs *etc*. and is not relevant to the choice of reflections for refinement. *R*-factors based on *F*^2^ are statistically about twice as large as those based on *F*, and *R*-factors based on ALL data will be even larger.

### Fractional atomic coordinates and isotropic or equivalent isotropic displacement parameters (Å^2^)

**Table:**

	*x*	*y*	*z*	*U*_iso_*/*U*_eq_	
S1	0.27208 (13)	0.30975 (11)	−0.03322 (9)	0.0585 (4)	
S9	0.44948 (13)	0.64057 (13)	0.38801 (8)	0.0588 (4)	
O23	0.1945 (3)	0.6524 (3)	0.7567 (2)	0.0571 (10)	
O25	−0.0298 (4)	0.8108 (3)	0.7090 (2)	0.0627 (10)	
N12	0.3407 (4)	0.6027 (3)	0.1072 (3)	0.0497 (10)	
C2	0.1374 (6)	0.1581 (4)	−0.0805 (4)	0.0608 (14)	
C3	0.0140 (6)	0.1630 (5)	−0.0311 (4)	0.0632 (16)	
C4	0.0329 (5)	0.2921 (4)	0.0500 (3)	0.0523 (12)	
C5	0.1673 (5)	0.3849 (4)	0.0577 (3)	0.0466 (11)	
C6	0.2239 (5)	0.5289 (4)	0.1278 (3)	0.0449 (11)	
C7	0.1393 (5)	0.5875 (4)	0.2153 (3)	0.0480 (11)	
C8	0.2257 (5)	0.5754 (4)	0.3433 (3)	0.0449 (11)	
C10	0.4599 (5)	0.7707 (4)	0.3043 (4)	0.0535 (12)	
C11	0.4061 (5)	0.7396 (4)	0.1762 (3)	0.0527 (12)	
C13	0.5351 (5)	0.9073 (5)	0.3660 (4)	0.0701 (17)	
C14	0.5566 (7)	1.0118 (5)	0.3038 (6)	0.083 (2)	
C15	0.5020 (7)	0.9803 (5)	0.1812 (6)	0.088 (2)	
C16	0.4288 (6)	0.8464 (5)	0.1171 (4)	0.0670 (17)	
C17	0.1517 (4)	0.6436 (4)	0.4359 (3)	0.0431 (11)	
C18	0.0340 (5)	0.7296 (4)	0.4125 (3)	0.0515 (13)	
C19	−0.0315 (5)	0.7881 (4)	0.5006 (3)	0.0556 (16)	
C20	0.0245 (5)	0.7594 (4)	0.6157 (3)	0.0482 (11)	
C21	0.1444 (5)	0.6730 (4)	0.6401 (3)	0.0444 (11)	
C22	0.2054 (4)	0.6162 (4)	0.5510 (3)	0.0413 (11)	
C24	0.3247 (6)	0.5738 (5)	0.7876 (3)	0.0632 (16)	
C26	−0.1737 (6)	0.8744 (6)	0.6826 (4)	0.0759 (19)	
H2	0.14610	0.07970	−0.13440	0.0730*	
H3	−0.07240	0.08940	−0.04890	0.0760*	
H4	−0.03800	0.31260	0.09370	0.0630*	
H7A	0.02720	0.53760	0.18850	0.0580*	
H7B	0.13320	0.68610	0.21410	0.0580*	
H8	0.21500	0.47440	0.34310	0.0540*	
H13	0.57160	0.92930	0.45010	0.0840*	
H14	0.60810	1.10260	0.34610	0.0990*	
H15	0.51440	1.05060	0.13970	0.1060*	
H16	0.39390	0.82690	0.03300	0.0800*	
H18	−0.00340	0.74950	0.33600	0.0620*	
H19	−0.11190	0.84580	0.48260	0.0660*	
H22	0.28500	0.55770	0.56850	0.0500*	
H24A	0.29120	0.47970	0.73860	0.0940*	
H24B	0.35240	0.57010	0.87140	0.0940*	
H24C	0.41930	0.61810	0.77380	0.0940*	
H26A	−0.15080	0.96270	0.66130	0.1140*	
H26B	−0.20870	0.89080	0.75250	0.1140*	
H26C	−0.25960	0.81310	0.61620	0.1140*	

### Atomic displacement parameters (Å^2^)

**Table:**

	*U*^11^	*U*^22^	*U*^33^	*U*^12^	*U*^13^	*U*^23^
S1	0.0629 (7)	0.0656 (7)	0.0458 (6)	0.0136 (5)	0.0226 (5)	−0.0012 (5)
S9	0.0514 (6)	0.0857 (8)	0.0389 (5)	0.0170 (5)	0.0149 (4)	0.0098 (5)
O23	0.0642 (18)	0.090 (2)	0.0267 (13)	0.0294 (15)	0.0199 (12)	0.0196 (13)
O25	0.0651 (18)	0.098 (2)	0.0329 (14)	0.0401 (16)	0.0224 (13)	0.0092 (14)
N12	0.0572 (19)	0.0571 (19)	0.0366 (16)	0.0108 (15)	0.0210 (15)	0.0044 (14)
C2	0.070 (3)	0.056 (2)	0.050 (2)	0.020 (2)	0.013 (2)	0.0028 (19)
C3	0.070 (3)	0.055 (2)	0.058 (3)	0.007 (2)	0.011 (2)	0.015 (2)
C4	0.058 (2)	0.062 (2)	0.040 (2)	0.0104 (19)	0.0177 (18)	0.0156 (18)
C5	0.053 (2)	0.056 (2)	0.0287 (17)	0.0129 (18)	0.0107 (16)	0.0070 (16)
C6	0.049 (2)	0.058 (2)	0.0291 (17)	0.0123 (17)	0.0148 (16)	0.0078 (16)
C7	0.052 (2)	0.061 (2)	0.0312 (18)	0.0097 (18)	0.0182 (16)	0.0020 (16)
C8	0.053 (2)	0.054 (2)	0.0299 (18)	0.0145 (17)	0.0174 (16)	0.0049 (16)
C10	0.048 (2)	0.063 (2)	0.049 (2)	0.0130 (19)	0.0210 (18)	0.0002 (19)
C11	0.054 (2)	0.061 (2)	0.047 (2)	0.0086 (19)	0.0264 (19)	0.0046 (19)
C13	0.055 (3)	0.074 (3)	0.067 (3)	0.007 (2)	0.019 (2)	−0.017 (2)
C14	0.087 (4)	0.060 (3)	0.102 (4)	0.004 (3)	0.046 (3)	−0.001 (3)
C15	0.099 (4)	0.057 (3)	0.132 (5)	0.011 (3)	0.074 (4)	0.020 (3)
C16	0.082 (3)	0.066 (3)	0.072 (3)	0.014 (2)	0.048 (3)	0.022 (2)
C17	0.048 (2)	0.051 (2)	0.0304 (18)	0.0061 (17)	0.0166 (15)	0.0035 (15)
C18	0.067 (3)	0.067 (2)	0.0273 (17)	0.026 (2)	0.0173 (17)	0.0169 (17)
C19	0.063 (3)	0.070 (3)	0.038 (2)	0.030 (2)	0.0177 (18)	0.0111 (18)
C20	0.053 (2)	0.062 (2)	0.0316 (19)	0.0152 (18)	0.0184 (16)	0.0042 (17)
C21	0.048 (2)	0.057 (2)	0.0303 (18)	0.0107 (17)	0.0154 (15)	0.0094 (16)
C22	0.048 (2)	0.054 (2)	0.0247 (17)	0.0152 (17)	0.0128 (15)	0.0107 (15)
C24	0.068 (3)	0.091 (3)	0.037 (2)	0.032 (2)	0.0165 (19)	0.022 (2)
C26	0.062 (3)	0.104 (4)	0.055 (3)	0.034 (3)	0.020 (2)	−0.011 (3)

### Geometric parameters (Å, º)

**Table:**

S1—C2	1.685 (5)	C17—C22	1.388 (5)
S1—C5	1.720 (4)	C18—C19	1.397 (6)
S9—C8	1.853 (5)	C19—C20	1.394 (5)
S9—C10	1.760 (4)	C20—C21	1.386 (6)
O23—C21	1.378 (4)	C21—C22	1.376 (5)
O23—C24	1.408 (6)	C2—H2	0.9300
O25—C20	1.366 (5)	C3—H3	0.9300
O25—C26	1.411 (6)	C4—H4	0.9300
N12—C6	1.294 (5)	C7—H7A	0.9700
N12—C11	1.400 (5)	C7—H7B	0.9700
C2—C3	1.365 (7)	C8—H8	0.9800
C3—C4	1.405 (6)	C13—H13	0.9300
C4—C5	1.373 (6)	C14—H14	0.9300
C5—C6	1.453 (5)	C15—H15	0.9300
C6—C7	1.507 (6)	C16—H16	0.9300
C7—C8	1.507 (5)	C18—H18	0.9300
C8—C17	1.520 (5)	C19—H19	0.9300
C10—C11	1.416 (6)	C22—H22	0.9300
C10—C13	1.391 (6)	C24—H24A	0.9600
C11—C16	1.388 (6)	C24—H24B	0.9600
C13—C14	1.393 (7)	C24—H24C	0.9600
C14—C15	1.355 (9)	C26—H26A	0.9600
C15—C16	1.372 (7)	C26—H26B	0.9600
C17—C18	1.368 (6)	C26—H26C	0.9600
			
C2—S1—C5	91.9 (2)	S1—C2—H2	124.00
C8—S9—C10	104.1 (2)	C3—C2—H2	124.00
C21—O23—C24	116.8 (3)	C2—C3—H3	124.00
C20—O25—C26	118.1 (3)	C4—C3—H3	124.00
C6—N12—C11	120.1 (3)	C3—C4—H4	124.00
S1—C2—C3	112.6 (3)	C5—C4—H4	124.00
C2—C3—C4	112.1 (4)	C6—C7—H7A	109.00
C3—C4—C5	112.7 (4)	C6—C7—H7B	109.00
S1—C5—C4	110.7 (3)	C8—C7—H7A	109.00
S1—C5—C6	120.3 (3)	C8—C7—H7B	109.00
C4—C5—C6	129.0 (4)	H7A—C7—H7B	108.00
N12—C6—C5	117.6 (4)	S9—C8—H8	107.00
N12—C6—C7	123.3 (3)	C7—C8—H8	107.00
C5—C6—C7	119.1 (4)	C17—C8—H8	107.00
C6—C7—C8	113.3 (3)	C10—C13—H13	120.00
S9—C8—C7	110.2 (3)	C14—C13—H13	119.00
S9—C8—C17	111.6 (2)	C13—C14—H14	120.00
C7—C8—C17	114.9 (3)	C15—C14—H14	120.00
S9—C10—C11	122.5 (3)	C14—C15—H15	119.00
S9—C10—C13	118.8 (3)	C16—C15—H15	119.00
C11—C10—C13	118.5 (4)	C11—C16—H16	120.00
N12—C11—C10	122.2 (3)	C15—C16—H16	120.00
N12—C11—C16	118.7 (3)	C17—C18—H18	119.00
C10—C11—C16	119.0 (4)	C19—C18—H18	119.00
C10—C13—C14	121.1 (4)	C18—C19—H19	120.00
C13—C14—C15	119.5 (5)	C20—C19—H19	120.00
C14—C15—C16	121.2 (5)	C17—C22—H22	119.00
C11—C16—C15	120.8 (4)	C21—C22—H22	119.00
C8—C17—C18	124.0 (3)	O23—C24—H24A	109.00
C8—C17—C22	118.2 (3)	O23—C24—H24B	109.00
C18—C17—C22	117.8 (3)	O23—C24—H24C	109.00
C17—C18—C19	121.7 (3)	H24A—C24—H24B	109.00
C18—C19—C20	119.6 (4)	H24A—C24—H24C	109.00
O25—C20—C19	124.8 (4)	H24B—C24—H24C	109.00
O25—C20—C21	116.2 (3)	O25—C26—H26A	110.00
C19—C20—C21	119.0 (4)	O25—C26—H26B	109.00
O23—C21—C20	115.1 (3)	O25—C26—H26C	109.00
O23—C21—C22	125.0 (4)	H26A—C26—H26B	109.00
C20—C21—C22	120.0 (3)	H26A—C26—H26C	109.00
C17—C22—C21	122.0 (4)	H26B—C26—H26C	109.00
			
C5—S1—C2—C3	0.9 (4)	S9—C8—C17—C22	−64.7 (4)
C2—S1—C5—C4	0.3 (3)	C7—C8—C17—C18	−10.4 (6)
C2—S1—C5—C6	−178.9 (3)	C7—C8—C17—C22	169.0 (3)
C10—S9—C8—C7	30.2 (3)	S9—C10—C11—N12	0.0 (6)
C10—S9—C8—C17	−98.7 (3)	S9—C10—C11—C16	−175.7 (4)
C8—S9—C10—C11	−62.9 (4)	C13—C10—C11—N12	175.1 (4)
C8—S9—C10—C13	122.1 (4)	C13—C10—C11—C16	−0.6 (7)
C24—O23—C21—C20	−175.5 (4)	S9—C10—C13—C14	175.6 (4)
C24—O23—C21—C22	3.9 (6)	C11—C10—C13—C14	0.3 (7)
C26—O25—C20—C19	13.0 (6)	N12—C11—C16—C15	−175.9 (5)
C26—O25—C20—C21	−167.2 (4)	C10—C11—C16—C15	−0.1 (8)
C11—N12—C6—C5	−176.7 (3)	C10—C13—C14—C15	0.6 (8)
C11—N12—C6—C7	6.6 (6)	C13—C14—C15—C16	−1.3 (9)
C6—N12—C11—C10	50.3 (6)	C14—C15—C16—C11	1.1 (9)
C6—N12—C11—C16	−134.0 (5)	C8—C17—C18—C19	179.1 (4)
S1—C2—C3—C4	−1.8 (5)	C22—C17—C18—C19	−0.3 (6)
C2—C3—C4—C5	2.1 (5)	C8—C17—C22—C21	−179.7 (4)
C3—C4—C5—S1	−1.4 (4)	C18—C17—C22—C21	−0.3 (6)
C3—C4—C5—C6	177.7 (4)	C17—C18—C19—C20	0.4 (6)
S1—C5—C6—N12	8.7 (5)	C18—C19—C20—O25	179.8 (4)
S1—C5—C6—C7	−174.4 (3)	C18—C19—C20—C21	0.0 (6)
C4—C5—C6—N12	−170.3 (4)	O25—C20—C21—O23	−0.9 (5)
C4—C5—C6—C7	6.5 (6)	O25—C20—C21—C22	179.7 (4)
N12—C6—C7—C8	−87.7 (5)	C19—C20—C21—O23	178.9 (4)
C5—C6—C7—C8	95.7 (4)	C19—C20—C21—C22	−0.5 (6)
C6—C7—C8—S9	48.4 (4)	O23—C21—C22—C17	−178.7 (4)
C6—C7—C8—C17	175.5 (3)	C20—C21—C22—C17	0.6 (6)
S9—C8—C17—C18	115.9 (4)		

### Hydrogen-bond geometry (Å, º)

**Table:**

*D*—H···*A*	*D*—H	H···*A*	*D*···*A*	*D*—H···*A*
C4—H4···O23^i^	0.93	2.54	3.457 (5)	169

Symmetry code: (i) −*x*, −*y*+1, −*z*+1.
